# 

**Published:** 1920-11-27

**Authors:** 


### Gifts and Bequests.

Mrs. Mary Radcliffe has bequeathed £500 to the women's ward of King Edward VII. Hospital, Cardiff.

Mrs. Hannah Ochiltree, of Newcastle-on-Tyne, under her will gives £5,000 to the Orthopædic Hospital, Newcastle, £1,000 to the Hospital for Sick Children, Moor Edge, and numerous other legacies to charities.

A cottage hospital at Alderley Edge, Cheshire, is to be founded by means of £20,000 from the estate of Mr. Otto Baerlein, formerly Servian Consul in Liverpool.

The Chelsea Hospital for Women has received from the Drapers' and Goldsmiths' Companies £50 each towards the new Nurses' Home.

King's College Hospital has received £100, duty free, under the will of the late Miss Etheland Lethbridge, £200 from Alexandra Rose Day Committee.

Mr. R. E. Pannett, of Whitby, solicitor, left £100 each to the Cottage Hospital and the Public Dispensary at Whitby.

Miss Edith Prance, of Frognal Lane, N.W., has left £50 each to the National Hospital for the Paralysed and Epileptic and University College Hospital.

Major-General C. E. Cumberland, of Maidstone, has left £50 each to the West Kent General Hospital and the Kent County Ophthalmic Hospital, Maidstone.

Mr. Thomas Whitaker, of Leeds, has left £100 to the Victoria Hospital, Keighley.

The Rev. James Butlin has left £50 each to the Warneford Hospital and the Midland Counties Home, Leamington.

Mrs. A. M. O. Bosher, of Middlesbrough, has left £100 each to the North Riding Infirmary and the Cottage Hospital.

The Works Hospital Committee of Messrs. J. S. White & Co., shipbuilders, have transferred £2,000 invested in National War Bonds to the Frank James Memorial Cottage Hospital at Cowes, to endow two beds.

The Great Northern Hospital has received a cheque for £715 13s. 4d. from the London County Council. This represents the sum earmarked by the contributory schools for the institution, and includes collections from elementary, private, commercial, technical, and secondary schools.

From the Alexandra Day Fund the Great Northern Central Hospital has received £500, the West London Hospital £300, the Kensington and Fulham General Hospital £250, the Seamen's Hospital, Greenwich, £200, Chelsea Hospital for Women £150, the Metropolitan Convalescent Institution, the London Orphan School, Watford, and the Homes for Little Boys, Farningham and Swanley, £100 each; and the Association for Promoting the Training and Supply of Midwives £50.

### Matrons' and Sisters' Appointments.

Fever Hospital, Kilsyth.—Miss Marion H. McIntyre has been appointed nurse-matron. She has been sister at Gordon Isolation Hospital and Sanatorium, and at Loanhead Isolation Hospital, Berwickshire, also at the Fever Hospitals, Oban and Linlithgow, and at Campbell Hospital, Portsoy.

Porth and District Hospital, Glamorgan.—Miss Frances Pratt has been appointed matron. She was trained at King Edward VII. Hospital, Cardiff, and holds the certificate of the Central Midwives Board. She has since been sister at Porth Hospital. Miss Pratt is a member of the College of Nursing. Miss Jennie Thomas has been appointed theatre sister. She was trained at the Royal Exeter and Devon Hospital, Exeter, where she was later staff nurse.

King's College Hospital, Denmark Hill.—Miss E. Cooke has been appointed home sister. She was trained at King's College Hospital, where she was later night sister. She has served for four and a-half years with the Territorial Force Nursing Service in France. Miss Annie Slater has been appointed sister. She was trained at King's College Hospital, and has since been *x*-ray sister at the Norfolk and Norwich Hospital.

Llwynpia Homes Infirmary.—Miss Esther Isaac has been appointed charge nurse. She was trained at Swansea General Hospital, and has since served with Queen Alexandra's Imperial Military Nursing Service Reserve in Mesopotamia. She has also done maternity work.

London Homœopathic Hospital, Great Ormond Street.—Miss Mabel Martin has been appointed sister. She was trained at the Middlesex Hospital, and has since been sister at the Royal Hospital, Richmond, Surrey, and at the Hospital for Women, Soho Square.

Wingrove Hospital, Newcastle.—Miss Annabella Baron, late assistant matron of the Wingrove Hospital under the Newcastle Board of Guardians, has been appointed matron. She became assistant matron in 1913, and served in Egypt from June 1915 to June 1919. Miss Baron was trained at Brentford Poor-law Infirmary.

Victoria Hospital, Blackpool.—Miss Edith M. Yeadon has been appointed host sister. She was trained at Portsmouth Royal Hospital, and holds the certificate of the Medico-Psychological Association. She has been sister-in-charge of the men's medical ward, surgical sister and sister-in-charge of the women's block at Canterbury General Hospital, sister-in-charge of the military block at Lincoln County Hospital and also night sister, night sister at Portsmouth Royal Hospital and sister-housekeeper. At Dorset County Mental Hospital she was night sister.

Royal National Hospital, Ventnor, I.O.W.—Mrs. Monteith has been appointed night sister. She was trained at Birmingham City Infirmary, and served throughout the war as sister at the 5th Southern General Hospital. She was awarded the Royal Red Cross. Previous to the war she was engaged in private nursing.

High Wycombe and Earl of Beaconsfield Memorial Cottage Hospital.—Miss Dorothy C. Philpott, A.R.R.C., has been appointed matron. She was trained at St. Thomas's Hospital and at the London Ophthalmic Hospital, and has since been sister at the West London Hospital, Hammersmith, theatre sister and assistant matron of Highfield Hall Red Cross Hospital, Southampton, and sister at St. John Brigade Hospital, Etaples.

Queen Charlotte's Hospital, Marylebone Road.—Miss Catharine A. Arkcoll has been appointed sister-midwife. She took her general training at the Miller General Hospital, and has been staff nurse at Queen Charlotte's Hospital, and now holds the post of sister of the Preliminary Training School. Miss Lucy B. Flemons has been appointed sister of one of the lying-in wards. She was trained at the Southwark Infirmary, and was staff nurse at the Samaritan Free Hospital and staff nurse at Queen Charlotte's Hospital.

